# Novel Effect of Zinc Nitrate/Vanadyl Oxalate for Selective Catalytic Oxidation of α-Hydroxy Esters to α-Keto Esters with Molecular Oxygen: An In Situ ATR-IR Study

**DOI:** 10.3390/molecules24071281

**Published:** 2019-04-02

**Authors:** Yongwei Ju, Zhongtian Du, Chuhong Xiao, Xingfei Li, Shuang Li

**Affiliations:** 1 School of Chemical Engineering, Northwest University, Xi’an 710069, China; 201620730@stumail.nwu.edu.cn; 2 School of Petroleum and Chemical Engineering, Dalian University of Technology, Panjin 124221, China; xiaochuhong@mail.dlut.edu.cn (C.X.); lixingfei620@mail.dlut.edu.cn (X.L.)

**Keywords:** ATR-IR, α-hydroxy esters, α-keto esters, oxidation, zinc nitrate

## Abstract

Selective oxidation of α-hydroxy esters is one of the most important methods to prepare high value-added α-keto esters. An efficient catalytic system consisting of Zn(NO_3_)_2_/VOC_2_O_4_ is reported for catalytic oxidation of α-hydroxy esters with molecular oxygen. Up to 99% conversion of methyl DL-mandelate or methyl lactate could be facilely obtained with high selectivity for its corresponding α-keto ester under mild reaction conditions. Zn(NO_3_)_2_ exhibited higher catalytic activity in combination with VOC_2_O_4_ compared with Fe(NO_3_)_3_ and different nitric oxidative gases were detected by situ attenuated total reflection infrared (ATR-IR) spectroscopy. UV-vis and ATR-IR results indicated that coordination complex formed in Zn(NO_3_)_2_ in CH_3_CN solution was quite different from Fe(NO_3_)_3_; it is proposed that the charge-transfer from Zn^2+^ to coordinated nitrate groups might account for the generation of different nitric oxidative gases. The XPS result indicate that nitric oxidative gas derived from the interaction of Zn(NO_3_)_2_ with VOC_2_O_4_ could be in favor of oxidizing VOC_2_O_4_ to generate active vanadium (V) species. It might account for different catalytic activity of Zn(NO_3_)_2_ or Fe(NO_3_)_3_ combined with VOC_2_O_4_. This work contributes to further development of efficient aerobic oxidation under mild reaction conditions.

## 1. Introduction

α-keto esters, which have two-functional groups, are valuable precursors in a variety of organic transformations [1,2,3]. For example, phenylglyoxylate and its derivatives are used as key intermediates for synthesis of herbicide metamitron [2], and pyruvate and its derivatives can be employed for the preparation of pharmaceutical pindolol and pesticides thiabendazole [3]. In the past several decades, several methods for synthesis of α-keto esters have been reported, such as esterification of α-keto acids or α-carbonyl aldehydes, oxidative cleavage of β-diketones, oxidative esterification of aryl-ketones and so on [4,5,6]. Considering the excellent atom efficiency, there is a growing interest to develop efficient catalytic oxidation of α-hydroxy esters into their corresponding α-keto esters [7,8].

Oxidation of α-hydroxy esters into α-keto esters is essentially selective oxidation of a secondary alcohol adjacent to a carboxylic ester group. Due to the inherent steric hindrance and electronic effect, complete conversion of α-hydroxy esters is usually difficult, while oxidative C-C bond cleavage could easily occur between two adjacent carbonyl carbon atoms under harsh oxidative reaction conditions [7,8]. Recently, several efficient aerobic catalytic systems have been developed under mild liquid reaction conditions [9,10,11,12,13], such as Pd-, Au-, and Ru-based catalysts, AZADO-NaNO_2_ and TEMPO-NaNO_2_. We are encouraged by all these methods, although some drawbacks still exist, such as side reactions, and use of expensive organic or metal catalysts. Therefore, it is still rather challenging for efficient catalytic oxidation of α-hydroxy esters into α-keto esters with molecular oxygen under mild reaction conditions.

As a base transitional metal, vanadium catalysts have been uncovered for aerobic oxidation of alcohols to carbonyl compounds recently [14,15]. Kirihara et al. achieved excellent yields of α-keto esters using fuming and corrosive VOCl_3_ [16]. Hanson et al. described the key role of pyridine during the alcohol oxidation by dipicolinate vanadium (V) [17,18]. Xu et al. also reported efficient catalytic oxidation of 5-hydroxymethylfurfural to furan-2,5-dicarbaldehyde, and C-C bond cleavage could be tuned using different vanadium-based catalysts [19,20]. These reports have provided us with important information concerning the selective oxidation of α-hydroxy esters using vanadium-based catalysts under mild reaction conditions. We used a highly efficient catalytic system consisting of Zn(NO_3_)_2_/VOC_2_O_4_ for selective oxidation of α-hydroxy esters into α-keto esters with molecular oxygen under mild reaction conditions, and employed infrared spectroscopy to investigate the unique catalytic activity of Zn(NO_3_)_2_ compared with other metal nitrates such as Fe(NO_3_)_3_.

## 2. Results and Discussion

Initially, oxidation of methyl DL-mandelate with molecular oxygen was selected as a model reaction, and performed in acetonitrile under mild reaction conditions (80 °C, 0.2 MPa O_2_). Table 1 shows the results on catalytic oxidation of methyl DL-mandelate with different catalysts. Only 5–6% conversion of methyl DL-mandelate was obtained using Zn(NO_3_)_2_ or VOC_2_O_4_ (Table 1, Entries 1–2). In contrast, 99% methyl phenylglyoxylate selectivity with 99% methyl DL-mandelate conversion was obtained within 1.5 h when both Zn(NO_3_)_2_ and VOC_2_O_4_ (molar ration = 1) were employed (Table 1, Entry 3). Moreover, no C-C bond cleavage of methyl phenylglyoxylate was observed even by prolonging the reaction time from 1.5 h to 6 h, which suggests that methyl phenylglyoxylate could remain stable under such oxidative environment (Table 1, Entry 4). Furthermore, nearly quantitative yield of methyl phenylglyoxylate could be facilely obtained at room temperature (25 °C) under normal pressure of molecular oxygen (Table 1, Entry 5). Compared with VOCl_3_ and other vanadium organic complexes [17,18,21,22,23], Zn(NO_3_)_2_/VOC_2_O_4_ is inexpensive and halogen free. All these results indicate that the catalytic system of Zn(NO_3_)_2_/VOC_2_O_4_ was efficient for catalytic oxidation of methyl DL-mandelate into methyl phenylglyoxylate with dioxygen under mild reaction conditions.

Further, we also compared different nitrates for catalytic oxidation of methyl DL-mandelate under the same reaction conditions (Table 1, Entries 6–10). When Co(NO_3_)_2_, Ce(NO_3_)_3_, Fe(NO_3_)_3_, Ni(NO_3_)_2_ and NaNO_3_ were employed instead of Zn(NO_3_)_2_, only 8–33% conversion of methyl DL-mandelate was observed. Zn(NO_3_)_2_ showed the best performance among those metal nitrates tested in this work. As a typical transition metal nitrate with multi-valences, Fe(NO_3_)_3_ was often used as a key component for selective oxidation of alcohols in previous reports [24,25]. Unexpectedly, Fe(NO_3_)_3_ displayed much lower catalytic activity than Zn(NO_3_)_2_, and only 16% methyl DL-mandelate conversion was obtained using Fe(NO_3_)_3_/VOC_2_O_4_ as the catalyst under the same reaction conditions. Thus, the nitrates could affect the catalytic activity significantly in this work. This issue is discussed in the following section.

Besides methyl DL-mandelate, lactate esters and benzoin were also oxidized (Table 2). Methyl lactate and ethyl lactate are typical aliphatic α-hydroxy esters, which are more difficult to be oxidized than methyl DL-mandelate. When they were oxidized, 21% and 59% conversion was obtained, respectively, with 99% pyruvate esters selectivity within 1.5 h. To our delight, 99% methyl lactate and ethyl lactate conversion with high selectivity of pyruvate esters was achieved after prolonging the reaction time to 4 h. Furthermore, good yields of pyruvate esters could also be attained at normal pressure of molecular oxygen at room temperature (Table 2, Entries 1–2). Moreover, the selectivity of byproducts (mainly acetic acid) derived from C-C bond cleavage was no more than 3%. As described above, it seemed difficult to obtain high yields of pyruvate directly from lactate via oxidation because of the side formation of CO_2_ through C−C bond fission [7,8]. For example, only 70% selectivity towards ethyl pyruvate was obtained when TiO_2_ was used for catalytic oxidation of ethyl lactate into ethyl pyruvate under mild reaction conditions [26]. Our results are competitive when compared with that using VOCl_3_ as a catalyst [27]. More than 95% benzil selectivity with 99% benzoin conversion could be achieved within 4 h. Oxidation of benzoin into benzil could also proceed smoothly under normal pressure of molecular oxygen at room temperature (Table 2, Entry 3). Only 5% benzoic acid/benzaldehyde byproducts were detected. Therefore, Zn(NO_3_)_2_/VOC_2_O_4_ is effective for catalytic oxidation of α-hydroxy compounds even under normal pressure and room temperature.

**Figure 1:**
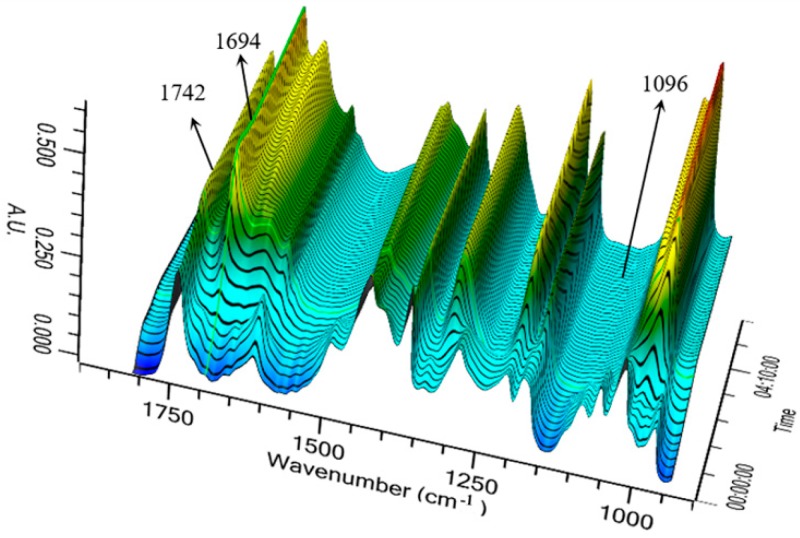
The real-time profile of methyl DL-mandelate oxidation monitored by online ATR-IR. Reaction conditions: 10 mmol methyl DL-mandelate, 1 mmol Zn(NO_3_)_2_, 1 mmol VOC_2_O_4_, 10 mL CH_3_CN, 80 °C, O_2_ balloon.

Operando attenuated total reflection infrared (ATR-IR) spectroscopy has been demonstrated as a powerful method to monitor real-time chemical reactions [28].To explore the oxidation process catalyzed by Zn(NO_3_)_2_/VOC_2_O_4_, oxidation of methyl DL-mandelate was monitored by online ATR-IR (Figure 1). The bands at 1694 and 1742 cm^−1^ were assigned to C=O stretching vibrations of carbonyl (methyl phenylglyoxylate) and esters functional group, respectively. The band at 1096 cm^−1^ was assigned to C-O stretching vibration of methyl DL-mandelate [29]. The signal at 1694 cm^−1^ rose gradually in the beginning, while the peak height at 1096 cm^−1^ (C-O stretching vibration of methyl DL-mandelate) diminished. Methyl DL-mandelate was converted to methyl phenylglyoxylate, and no other significant intermediates were detected by ATR-IR during the oxidation reaction. Moreover, the peaks at 1742 and 1694 cm^−1^ remained stable as the reaction time prolonged, and no other significant signals appeared, indicating that few byproducts were generated under such oxidative conditions. These observations were in accordance with GC analysis (Table 1).

**Figure 2:**
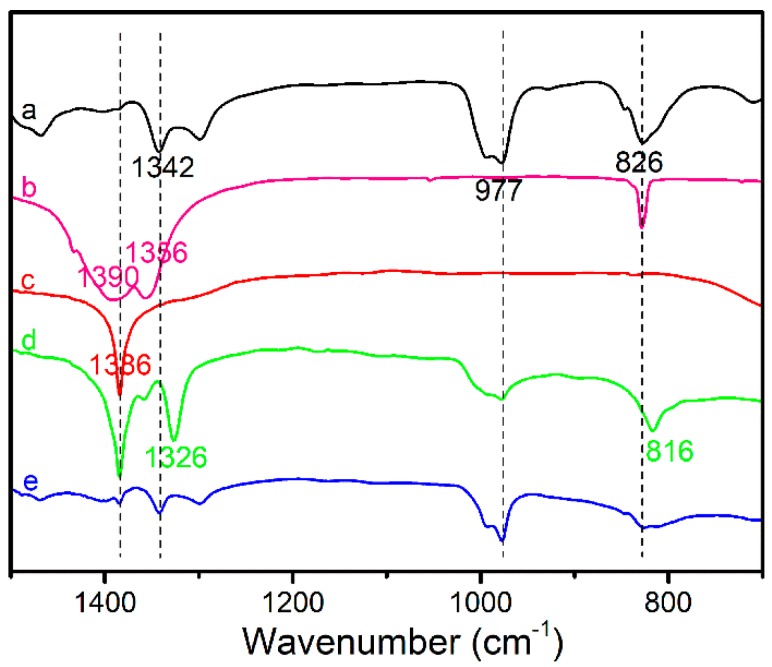
FT-IR investigation on the interaction of nitrates and VOC_2_O_4_: (a) VOC_2_O_4_; (b) Zn(NO_3_)_2_; (c) Fe(NO_3_)_3_; (d) Zn(NO_3_)_2_ + VOC_2_O_4_, Zn(NO_3_)_2_:VOC_2_O_4_ = 1:1 (molar ratio), 0.2 MPa O_2_, 5 mL CH_3_CN, 80 °C, 1.5 h; and (e) Fe(NO_3_)_3_ + VOC_2_O_4_, Fe(NO_3_)_3_:VOC_2_O_4_ = 1:1 (molar ratio), 0.2 MPa O_2_, 5 mL CH_3_CN, 80 °C, 1.5 h.

To explore the different catalytic activities of nitrates, we selected Zn(NO_3_)_2_ and Fe(NO_3_)_3_ to study the interaction of catalyst components using FT-IR. The reaction of Zn(NO_3_)_2_ or Fe(NO_3_)_3_ with VOC_2_O_4_ was carried out in acetonitrile without methyl DL-mandelate (80 °C, 0.2 MPa O_2_, 1.5 h). After the pre-treatment, a red brown powder was obtained after removing acetonitrile. The FT-IR spectra are presented in Figure 2. For the FT-IR spectrum of VOC_2_O_4_ (Figure 2, Line a), the band at 977 cm^−1^ was attributed to be characteristic of V=O stretching vibration and the 826 cm^−1^ band was due to O-C=C bending and M-O stretching vibration [30]. The shoulder bands at 1390 and 1356 cm^−1^ were assigned to N-O stretching vibrations of Zn(NO_3_)_2_ (Figure 2, Line b), while the sharp band at 1386 cm^−1^ was attributed to N-O stretching vibration of Fe(NO_3_)_3_ (Figure 2, Line c) [31]. It should be noted that the FT-IR spectrum of reaction mixture derived from Zn(NO_3_)_2_/VOC_2_O_4_ (Figure 2, Line d) was quite different from those of VOC_2_O_4_ or Zn(NO_3_)_2_ (Figure 2, Lines a and b). Two new bands at 1326 and 816 cm^−1^ (Figure 2, Line d) appeared while no similar new absorption bands were generated when VOC_2_O_4_ was reacted with Fe(NO_3_)_3_ under the same reaction conditions (Figure 2, Line e). These results suggest that the reaction mixtures derived from Zn(NO_3_)_2_ and Fe(NO_3_)_3_/VOC_2_O_4_ were quite different, especially the form of nitric species. These phenomena may be related to the generation of different NO_x_ gases, which might account for catalytic activity difference by using Zn(NO_3_)_2_ or Fe(NO_3_)_3_ with VOC_2_O_4_, respectively.

**Figure 3:**
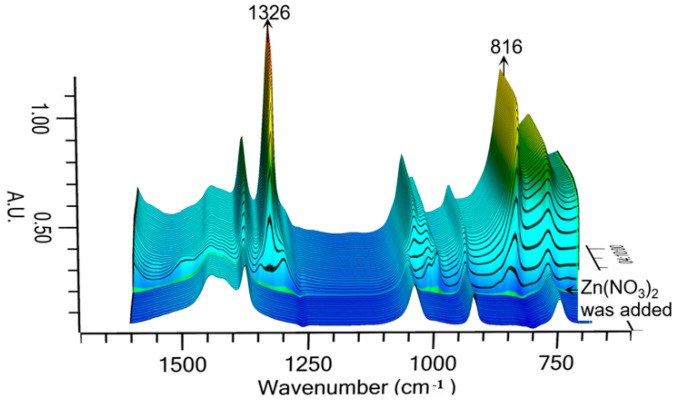

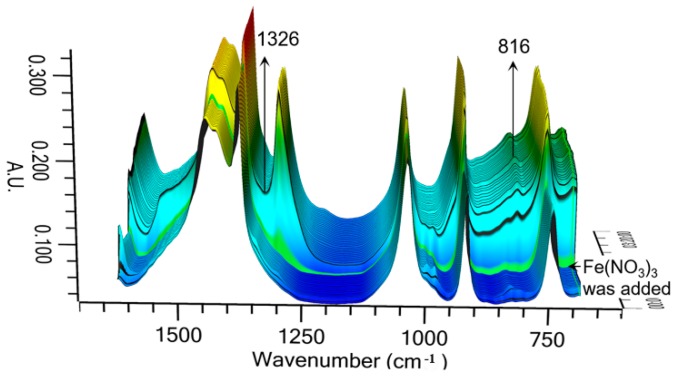
In situ ATR-IR investigation on interaction of nitrates and VOC_2_O_4_, nitrates:VOC_2_O_4_ = 1:1 (molar ratio): Interaction of Zn(NO_3_)_2_ and VOC_2_O_4_ (**top**); and interaction of Fe(NO_3_)_3_ and VOC_2_O_4_ (**bottom**).

The observation above was further confirmed by investigating the interaction of Zn(NO_3_)_2_ or Fe(NO_3_)_3_ with VOC_2_O_4_ using online ATR-IR (Figure 3). Firstly, in the absence of substrate, VOC_2_O_4_ was stirred at 80 °C in acetonitrile under an oxygen atmosphere, and no apparent variation of spectrum was observed. This observation suggests that VOC_2_O_4_ alone remained stable under oxidative reaction conditions. When Zn(NO_3_)_2_ was introduced, new bands at 1326 and 816 cm^−1^ were generated simultaneously (Figure 3, top). On the contrary, when Fe(NO_3_)_3_ was added under the same reaction conditions, the ATR-IR spectrum (Figure 3, bottom) was distinct from that derived from Zn(NO_3_)_2_/VOC_2_O_4_. The bands at 1326 and 816 cm^−1^ were not observed (Figure 3, bottom). The online ATR-IR results were in good agreement with FT-IR results (Figure 2). These results demonstrated that different nitric species occurred in liquid phase by adding Zn(NO_3_)_2_ or Fe(NO_3_)_3_ under such oxidative conditions. This difference might be associated with the generation of NO_x_ gases. It might account for lower catalytic activity for Fe(NO_3_)_3_ than that of Zn(NO_3_)_2_.

**Figure 4:**
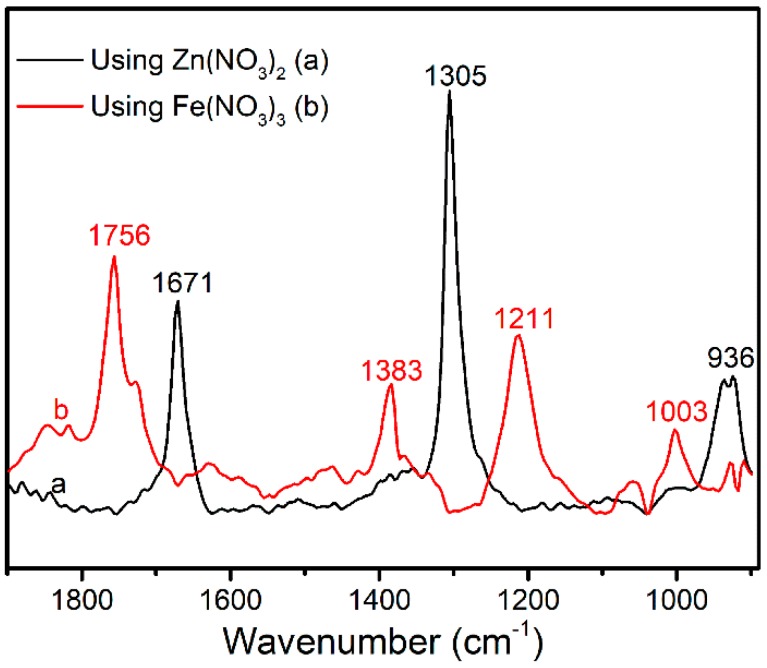
NO_x_ gas detected by in situ ATR-IR. Reaction conditions: 1 mmol Zn(NO_3_)_2_ or Fe(NO_3_)_3_, 1 mmol VOC_2_O_4_, 10 mL CH_3_CN, 80 °C, O_2_ balloon.

To verify the hypothesis mentioned above, we employed ATR-IR spectroscopy to detect composition of the gas phase, which was generated from the reaction of Zn(NO_3_)_2_/VOC_2_O_4_ or Fe(NO_3_)_3_/VOC_2_O_4_ (Figure 4). Firstly, Zn(NO_3_)_2_/VOC_2_O_4_ was stirred without the substrate under an oxygen atmosphere at 80 °C. In these experiments, the React IR™ 15-diamond probe was immersed in gas phase rather than liquid phase. The ATR-IR spectrum of gas phase demonstrated that new species which IR bands at 1671, 1305 and 936 cm^−1^ generated were attributable to HNO_3_ vapor (Figure 4, Line a) [32]. This observation suggests that HNO_3_ vapor probably was involved as one of key components during the oxidation using Zn(NO_3_)_2_/VOC_2_O_4_. In contrast, when the gas derived from Fe(NO_3_)_3_/VOC_2_O_4_ was monitored under the same conditions, IR bands at 1756, 1383, 1211 and 1003 cm^−1^ were detected. These bands were related to N_2_O_3_ and N_2_O_4_ mixture gas (Figure 4, Line b) [33,34], which were quite different from those derived from Zn(NO_3_)_2_/VOC_2_O_4_ (Figure 4, Line a). All these results above indicate that different NO_x_ gases were involved during catalytic oxidation using Zn(NO_3_)_2_/VOC_2_O_4_ or Fe(NO_3_)_3_/VOC_2_O_4_. The obvious difference in NO_x_ gases generated by using Zn(NO_3_)_2_/VOC_2_O_4_ or Fe(NO_3_)_3_/VOC_2_O_4_ was further investigated.

UV-vis spectroscopy was employed to detect coordination state of Zn(NO_3_)_2_/Fe(NO_3_)_3_ in CH_3_CN. An intense and broad peak at 293 nm was observed in Zn(NO_3_)_2_ in CH_3_CN solution, which was assigned to the n–π* transition, while no obvious peak was found on Fe(NO_3_)_3_ in CH_3_CN solution (Figure S2). It is proposed that the observed variation in the UV-vis spectrum should be interpreted as a coordination interaction taking place between Zn(NO_3_)_2_ and CH_3_CN solvent [35]. ATR-IR spectra in the C-N region of Zn(NO_3_)_2_/Fe(NO_3_)_3_ in CH_3_CN solution are shown in Figure S3. The bands at 2253 and 2292 cm^−1^ were assigned to C-N stretching vibrations of CH_3_CN, a new band at 2314 cm^−1^ appeared as Zn(NO_3_)_2_ was added to CH_3_CN, while no new band at 2314 cm^−1^ appeared as Fe(NO_3_)_3_ was added to CH_3_CN. This difference could be attributed to the unique complete filled 3d electronic orbitals of Zn^2+^ (3d^10^), which is quite different from Fe^3+^ (3d^5^) causing vibrations of the coordinated CH_3_CN. Previous studies also report a similar [Zn(CH_3_CN)_2_](NO_3_)_2_ coordination complex forming in Zn(NO_3_)_2_ in CH_3_CN solvent [36]. It can be inferred that charge-transfer from Zn^2+^ to coordinated nitrate groups might account for the generation of different NO_x_ gases using Zn(NO_3_)_2_ and Fe(NO_3_)_3_, respectively [37].

**Figure 5:**
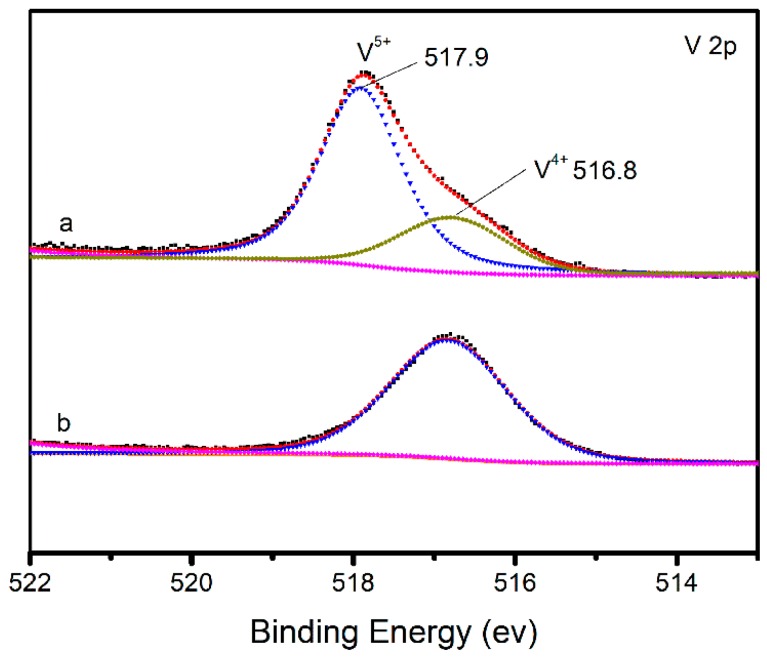
V 2p XPS spectra of the reaction mixture: V 2p XPS spectra of the Zn(NO_3_)_2_/VOC_2_O_4_ reaction mixture (a); and V 2p XPS spectra of the Fe(NO_3_)_3_/VOC_2_O_4_ reaction mixture (b).

The catalytic system consisting of nitrates and vanadium compounds has been reported, and the cascade electron transfer among nitric, vanadium species and oxygen was proposed [19,20]. vanadium (IV) species could be easily oxidized to vanadium (V) species by nitric oxidative gas rather than oxygen and after that the active vanadium (V) species oxidized alcohol group to ketone. We also believe that similar redox cycles existed in this work. To verify whether this nitric oxidative gas derived from the interaction of Zn(NO_3_)_2_ with VOC_2_O_4_ could promote the oxidation of VOC_2_O_4_ to generate active vanadium (V) species. XPS measurements were employed to detect the active vanadium (V) species after the interaction of Zn(NO_3_)_2_ or Fe(NO_3_)_3_ with VOC_2_O_4_. Firstly, the reaction of Zn(NO_3_)_2_ or Fe(NO_3_)_3_ with VOC_2_O_4_ was carried out in acetonitrile (80 °C, 0.2 MPa O_2_, 1.5 h). The reaction mixture was then obtained after removing acetonitrile. V 2p XPS spectra of the reaction mixture was shown in Figure 5. The peak of vanadium (IV) locates at 516.8 eV and that of vanadium (V) at 517.9 eV, which are in good accordance with previous report (516.5 eV for vanadium (IV) and 517.6 eV for vanadium (V)) [38]. It can be seen that vanadium (V) species appeared after the interaction of Zn(NO_3_)_2_ with VOC_2_O_4_ (Figure 5, Line a), while no vanadium (V) species existed in the reaction mixture of Fe(NO_3_)_3_/VOC_2_O_4_ under the same reaction conditions (Figure 5, Line b). V 2p XPS spectra of the reaction mixture provided strong evidence for the generation of vanadium (V) species in the Zn(NO_3_)_2_/VOC_2_O_4_ catalytic system. It is known that oxidizing ability of nitric oxidative gas was quite different [39]. Combining ATR-IR with XPS characterization results suggests that HNO_3_ gas derived from the interaction of Zn(NO_3_)_2_ with VOC_2_O_4_ could be in favor of oxidizing vanadium (IV) species to active vanadium (V) species, thus explaining why Zn(NO_3_)_2_ combined with VOC_2_O_4_ exhibited unique catalytic activity, compared with other tested metal nitrates such as Fe(NO_3_)_3_ under the same reaction conditions.

**Scheme 1:**
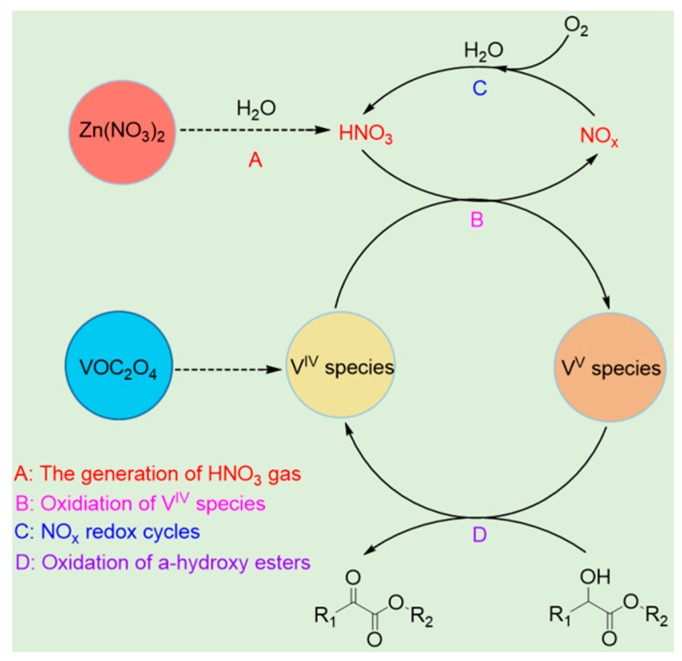
Plausible reaction mechanism for Zn(NO_3_)_2_/VOC_2_O_4_ catalyzed aerobic oxidation of α-hydroxy esters.

Based on the above results and previous reports [19,20], the reaction mechanism for Zn(NO_3_)_2_/VOC_2_O_4_ catalyzed aerobic oxidation of α-hydroxy esters is proposed in Scheme 1. Firstly, the interaction of VOC_2_O_4_ and Zn(NO_3_)_2_ occurred under such oxidative conditions. In the presence of water, the oxidative gas of HNO_3_ was generated after the interaction of VOC_2_O_4_ with Zn(NO_3_)_2_. Then, vanadium (IV) species derived from VOC_2_O_4_ underwent oxidation to vanadium (V) species by HNO_3_ gas, and after that the active vanadium (V) species oxidized α-hydroxy esters to α-keto esters with the formation of vanadium (IV) species. In this oxidation reaction, the oxidative gas of HNO_3_ generated in this catalyst system is expected to initiate and sustain vanadium (IV)/vanadium (V) redox cycle, and molecular oxygen plays an important role in NO_x_ redox cycle. Such proposed reaction mechanism could explain the above experimental phenomena.

## 3. Materials and Methods

### 3.1. Catalyst Preparation

VOC_2_O_4_·2H_2_O was synthesized according to literature procedure [40]. Typically, vanadium pentoxide (2.0 g), oxalic acid dihydrate (4.3 g) and glacial acetic acid (16.8 mL) were added to a flask (50 mL). The reaction mixture was stirred for 2 h at 110 °C. The resulting slurry was cooled to room temperature, filtered, and the filtrate was discarded. The solid was dried at 80 °C under a vacuum for 5 h. The FT-IR spectrum of VOC_2_O_4_·2H_2_O is presented in Figure S1.

### 3.2. Typical Procedures of Reaction

In a typical procedure for catalytic oxidation α-hydroxy esters, α-hydroxy esters (5 mmol), VOC_2_O_4_·2H_2_O (0.25 mmol), Zn(NO_3_)_2_·6H_2_O (0.25 mmol), and CH_3_CN (5 mL) were placed in autoclave (25 mL). Oxygen was then added (0.2 MPa). The mixture was stirred at 80 °C for 1.5 h. After the reaction was completed, the resulting mixture was filtered and determined by gas chromatography (GC-7900 equipped with FID detector and PEG-20 capillary column, Techcomp Ltd, Shanghai, China) Conversion and selectivity were determined based on area normalization method. Products were verified by GC-MS, NMR and ATR-IR.

### 3.3. Typical Procedures of Analysis Method

GC-MS was performed on a Thermo Fisher Trace 1300s-ISQ LT instrument (Thermo Fisher, Waltham, MA, USA) with electron ionization (EI) mass spectrometry. NMR spectra were recorded at 500 MHz with Bruker Avance III HD instrument (Bruker, Billerica, MA, USA) using CDCl_3_ as the solvent with tetramethylsilane (TMS) as the internal standard.

The FT-IR spectra were recorded within a 4000–500 cm^−1^ region on a Thermo Fisher Nicolet iN10 MX & iS10 infrared spectrometer (Thermo Fisher, Waltham, MA, USA) using KBr pellets.

The UV–vis spectra was recorded within a 200–800 nm region on an Agilent Cary 60 spectrophotometer (Agilent, Palo Alto, CA, USA) with quartz cells at room temperature.

XPS measurements were performed on a Thermo Fisher ESCALAB 250Xi spectrometer (Thermo Fisher, Waltham, MA, USA).

The situ ATR-IR spectra were recorded within a 3000–600 cm^−1^ region on a Mettler Toledo React IR15 spectrometer (Mettler Toledo, Zurich, Switzerland) equipped with a liquid-nitrogen-cooled MCT (mercury cadmium telluride) detector and a diamond probe. The typical experiments were carried out in a closed flask. The IR probe was immersed in liquid phase reaction mixture or gas phase.

## 4. Conclusions

An efficient catalytic system consisting of Zn(NO_3_)_2_/VOC_2_O_4_ was developed for aerobic oxidation of α-hydroxy esters. Methyl DL-mandelate or methyl lactate could be facilely oxidized into its corresponding α-keto ester with high yields (up to 99% conversion of α-hydroxy esters) even under normal pressure at room temperature. Zn(NO_3_)_2_ combined with VOC_2_O_4_ exhibited unique catalytic activity compared to other tested metal nitrates such as Fe(NO_3_)_3_. Different nitric species were generated after interaction of VOC_2_O_4_ with Zn(NO_3_)_2_ or Fe(NO_3_)_3_. The coordination complex was identified by UV-vis and ATR-IR indicated the unique reaction route in Zn(NO_3_)_2_/VOC_2_O_4_, which gave diverse nitric oxidative gas with different oxidizing ability. The XPS result suggested that HNO_3_ gas derived from the interaction of Zn(NO_3_)_2_ with VOC_2_O_4_ could promote the oxidation of VOC_2_O_4_ to generate active vanadium (V) species. Further development of efficient vanadium-based catalysts for oxidation of alcohols is currently underway.

## Figures and Tables

**Table 1:**
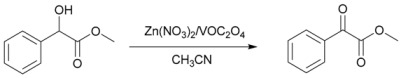
Catalytic oxidation of methyl DL-mandelate with different catalyst ^a^.

Entry	Metal Nitrate	t (h)	Conversion (%)	Selectivity ^b^ (%)
1	-	1.5	6	>99
2 ^c^	Zn(NO_3_)_2_	1.5	5	>99
3	Zn(NO_3_)_2_	1.5	>99	>99
4	Zn(NO_3_)_2_	6	>99	>99
5 ^d^	Zn(NO_3_)_2_	48	>99	>99
6	NaNO_3_	1.5	33	95
7	Ni(NO_3_)_2_	1.5	25	>99
8	Fe(NO_3_)_3_	1.5	16	95
9	Ce(NO_3_)_3_	1.5	11	>99
10	Co(NO_3_)_2_	1.5	8	>99

^a^ Reaction conditions: 5 mmol methyl DL-mandelate, 0.25 mmol metal nitrate, 0.25 mmol VOC_2_O_4_, 5 mL CH_3_CN, 80 °C, 0.2 MPa O_2_, 1.5 h. ^b^ Selectivity toward for methyl phenylglyoxylate. ^c^ No VOC_2_O_4_ was added. ^d^ Room temperature, normal pressure using O_2_ balloon.

**Table 2:** Catalytic oxidation of lactate esters and benzoin with Zn(NO_3_)_2_/VOC_2_O_4_^a^.

Entry	Substrate	Product	t (h)	Conversion (%)	Selectivity (%)
1	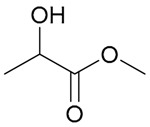	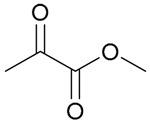	1.5	59	99
			4	>99	97
			72 ^b^	>99	97
2	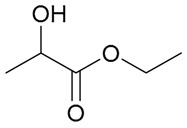	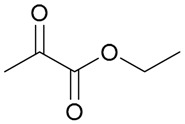	1.5	21	99
			4	>99	98
			72 ^b^	>99	98
3	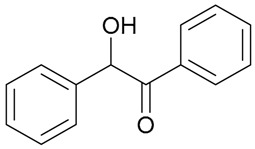	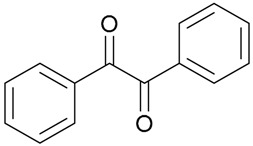	1.5	81	96
			4	>99	95
			48 ^b^	>99	95

^a^ Reaction conditions: 5 mmol substrate, 0.25 mmol Zn(NO_3_)_2_, 0.25 mmol VOC_2_O_4_, 5 mL CH_3_CN, 80 °C, 0.2 MPa O_2_. ^b^ Room temperature, normal pressure using O_2_ balloon.

## Supplementary Materials

The supplementary materials are available online, Figure S1: FT-IR spectrum of vanadyl oxalate, Figure S2: UV–vis spectra of Zn(NO_3_)_2_ or Fe(NO_3_)_3_ in CH_3_CN solvent, Figure S3: ATR-IR spectra in the C-N region of Zn(NO_3_)_2_ or Fe(NO_3_)_3_ in CH_3_CN solvent, Figures S4 and S5: NMR spectra of products, Figures S6–S9: MS spectra of products, Figures S10–S17: Original spectra of GC. Table S1: The effect of catalyst loading and Zn(NO_3_)_2_ loading for catalytic oxidation of methyl DL-mandelate, Table S2: Catalytic oxidation of various alcohols.
