# Supplementary material for: Novel Effect of Zinc Nitrate/Vanadyl Oxalate for Selective Catalytic Oxidation of α-Hydroxy Esters to α-Keto Esters with Molecular Oxygen: An In Situ ATR-IR Study
Source: Molecules. 2019 Apr 2;24(7):1281. doi: 10.3390/molecules24071281 (PMC6480123; doi:10.3390/molecules24071281)
Supplement: Supplementary file 1 [file molecules-24-01281-s001.pdf]

Supporting Information

Novel Effect of Zinc Nitrate/Vanadyl Oxalate for Selective Catalytic Oxidation of α-Hydroxy Esters to α-Keto Esters with Molecular Oxygen: An In Situ ATR-IR Study

Yongwei Ju [1], Zhongtian Du [2,*], Chuhong Xiao [2], Xingfei Li [2] and Shuang Li [1,*]

[1] School of Chemical Engineering, Northwest University, Xi'an 710069, China; 201620730@stumail.nwu.edu.cn

[2] School of Petroleum and Chemical Engineering, Dalian University of Technology, Panjin 124221, China; xiaochuhong@mail.dlut.edu.cn (C.X.); lixingfei620@mail.dlut.edu.cn (X.L.)

* Correspondence: duzhongtian@dlut.edu.cn (Z.D.); shuangli722@126.com (S.L.); Tel.: +86-029-88303733 (S.L.)

Contents:

1. **Typical procedures of reaction (pp.2-4)**
2. **FT-IR spectrum of vanadyl oxalate (p.4)**
3. **UV–vis spectra of $Zn(NO_3)_2$ or $Fe(NO_3)_3$ in CH_3CN solvent (p.4)**
4. **ATR-IR spectra in the C-N region of $Zn(NO_3)_2$ or $Fe(NO_3)_3$ in CH_3CN solvent (p.5)**
5. **The effect of catalyst loading and $Zn(NO_3)_2$ loading for catalytic oxidation of methyl DL-mandelate (pp.5-6)**
6. **Catalytic oxidation of various alcohols (pp.6-7)**
7. **NMR and MS spectra (pp.8-11)**
8. **Original spectra of GC (pp.12-15)**

1. Typical procedures of reaction

General procedure for synthesis of methyl phenylglyoxylate: Methyl DL-mandelate (5 mmol), $VOC_2O_4·2H_2O$ (0.25 mmol), $Zn(NO_3)_2·6H_2O$ (0.25 mmol), and CH_3CN (5 mL) were placed in autoclave (25 mL). After the autoclave was closed, oxygen was added (0.2 MPa). The mixture was stirred at 80 °C for 1.5 h. After the reaction was completed, the autoclave was cooled to room temperature. The resulting mixture was filtered, the organic phase was concentrated under reduced pressure, and the crude product was purified by silica gel column chromatography (petroleum ether/ethyl acetate 20:1). ^{1}H NMR (500 MHz, $CDCl_3$) δ = 8.02 (d, *J* = 7.2 Hz, 2H), 7.67 (t, *J* = 7.5 Hz, 1H), 7.52 (t, *J* = 7.8 Hz, 2H), 3.98 (s, 3H). ^{13}C NMR (126 MHz, $CDCl_3$) δ = 186.25, 164.24, 135.20, 132.63, 130.30, 129.12, 52.99. GC/MS (EI, 70 eV) m/z: 164.15.

General procedure for synthesis of methyl pyruvate: Methyl lactate (5 mmol), $VOC_2O_4·2H_2O$ (0.25 mmol), $Zn(NO_3)_2·6H_2O$ (0.25 mmol), and CH_3CN (5 mL) were placed in autoclave (25 mL). After the autoclave was closed, oxygen was added (0.2 MPa). The mixture was stirred at 80 °C for 4 h. After the reaction was completed, the autoclave was cooled to room temperature. The resulting mixture was filtered and determined by GC and GC-MS. GC/MS (EI, 70 eV) m/z: 102.09.

General procedure for synthesis of ethyl pyruvate: Ethyl lactate (5 mmol), $VOC_2O_4·2H_2O$ (0.25 mmol), $Zn(NO_3)_2·6H_2O$ (0.25 mmol), and CH_3CN (5 mL) were placed in autoclave (25 mL). After the autoclave was closed, oxygen was added (0.2 MPa). The mixture was stirred at 80 °C for 4 h. After the reaction was completed, the autoclave was cooled to room temperature. The resulting mixture was filtered and determined by GC and GC-MS. GC/MS (EI, 70 eV) m/z: 116.11.

General procedure for synthesis of benzil: Benzoin (5 mmol), $VOC_2O_4·2H_2O$ (0.25 mmol), $Zn(NO_3)_2·6H_2O$ (0.25 mmol), and CH_3CN (5 mL) were placed in autoclave (25 mL). After the autoclave was closed, oxygen was added (0.2 MPa). The mixture was stirred at 80 °C for 4 h. After the reaction was completed, the autoclave was cooled to room temperature. The resulting mixture was filtered, the organic phase was concentrated under reduced pressure, and the crude product was purified by silica gel column chromatography (petroleum ether/ethyl acetate 20:1). ^{1}H NMR (500 MHz, $CDCl_3$) δ = 7.98 (m, 4H), 7.66 (t, *J* = 7.5 Hz, 2H), 7.52 (t, *J* = 7.8 Hz, 4H). ^{13}C NMR (126 MHz, $CDCl_3$) δ = 194.78, 135.10, 133.19, 130.11, 129.23. GC/MS (EI, 70 eV) m/z: 210.20.

General procedure for synthesis of benzaldehyde: Benzyl alcohol (5 mmol), $VOC_2O_4·2H_2O$ (0.25 mmol), $Zn(NO_3)_2·6H_2O$ (0.25 mmol), and CH_3CN (5 mL) were placed in autoclave (25 mL). After

the autoclave was closed, oxygen was added (0.2 MPa). The mixture was stirred at 80 °C for 4 h. After the reaction was completed, the autoclave was cooled to room temperature. The resulting mixture was determined by GC and comparison with the authentic samples.

General procedure for synthesis of 4-nitrobenzaldehyde: 4-Nitrobenzyl alcohol (5 mmol), $VOC_2O_4\cdot 2H_2O$ (0.25 mmol), $Zn(NO_3)_2\cdot 6H_2O$ (0.25 mmol), and CH_3CN (5 mL) were placed in autoclave (25 mL). After the autoclave was closed, oxygen was added (0.2 MPa). The mixture was stirred at 80 °C for 4 h. After the reaction was completed, the autoclave was cooled to room temperature. The resulting mixture was determined by GC and comparison with the authentic samples.

General procedure for synthesis of 2-thenaldehyde: 2-Thiophenemethanol (5 mmol), $VOC_2O_4\cdot 2H_2O$ (0.25 mmol), $Zn(NO_3)_2\cdot 6H_2O$ (0.25 mmol), and CH_3CN (5 mL) were placed in autoclave (25 mL). After the autoclave was closed, oxygen was added (0.2 MPa). The mixture was stirred at 80 °C for 1.5 h. After the reaction was completed, the autoclave was cooled to room temperature. The resulting mixture was determined by GC and comparison with the authentic samples.

General procedure for synthesis of 2-pyridinecarboxaldehyde: 2-Pyridinemethanol (5 mmol), $VOC_2O_4\cdot 2H_2O$ (0.1 mmol), $Zn(NO_3)_2\cdot 6H_2O$ (0.1 mmol), and CH_3CN (5 mL) were placed in autoclave (25 mL). After the autoclave was closed, oxygen was added (0.2 MPa). The mixture was stirred at 80 °C for 1.5 h. After the reaction was completed, the autoclave was cooled to room temperature. The resulting mixture was determined by GC and comparison with the authentic samples.

General procedure for synthesis of acetophenone: Phenylethyl alcohol (5 mmol), $VOC_2O_4\cdot 2H_2O$ (0.25 mmol), $Zn(NO_3)_2\cdot 6H_2O$ (0.25 mmol), and CH_3CN (5 mL) were placed in autoclave (25 mL). After the autoclave was closed, oxygen was added (0.2 MPa). The mixture was stirred at 80 °C for 12 h. After the reaction was completed, the autoclave was cooled to room temperature. The resulting mixture was determined by GC and comparison with the authentic samples.

General procedure for synthesis of 3-pyridinecarboxaldehyde: 3-Pyridinemethanol (5 mmol), $VOC_2O_4\cdot 2H_2O$ (0.25 mmol), $Zn(NO_3)_2\cdot 6H_2O$ (0.25 mmol), and CH_3CN (5 mL) were placed in autoclave (25 mL). After the autoclave was closed, oxygen was added (0.2 MPa). The mixture was stirred at 80 °C for 6 h. After the reaction was completed, the autoclave was cooled to room temperature. The resulting mixture was determined by GC and comparison with the authentic samples.

General procedure for synthesis of 2-pentanone: 2-Amyl alcohol (5 mmol), $VOC_2O_4\cdot 2H_2O$ (0.25 mmol), $Zn(NO_3)_2\cdot 6H_2O$ (0.25 mmol), and CH_3CN (5 mL) were placed in autoclave (25 mL). After the

autoclave was closed, oxygen was added (0.2 MPa). The mixture was stirred at 80 °C for 12 h. After the reaction was completed, the autoclave was cooled to room temperature. The resulting mixture was determined by GC and comparison with the authentic samples.

General procedure for synthesis of cyclohexanone: Cyclohexanol (5 mmol), $VOC_2O_4·2H_2O$ (0.25 mmol), $Zn(NO_3)_2·6H_2O$ (0.25 mmol), and CH_3CN (5 mL) were placed in autoclave (25 mL). After the autoclave was closed, oxygen was added (0.2 MPa). The mixture was stirred at 80 °C for 12 h. After the reaction was completed, the autoclave was cooled to room temperature. The resulting mixture was determined by GC and comparison with the authentic samples.

2. FT-IR spectrum of vanadyl oxalate

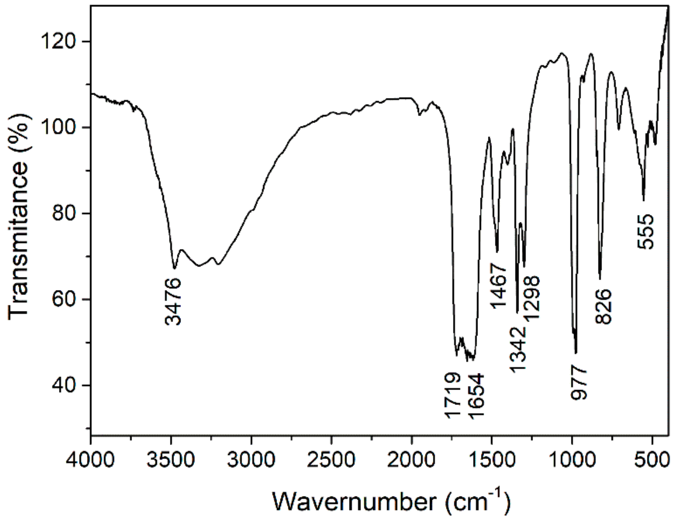

Figure S1. The FT-IR spectrum of $VOC_2O_4·2H_2O$.

3. UV–vis spectra of $Zn(NO_3)_2$ or $Fe(NO_3)_3$ in CH_3CN solvent

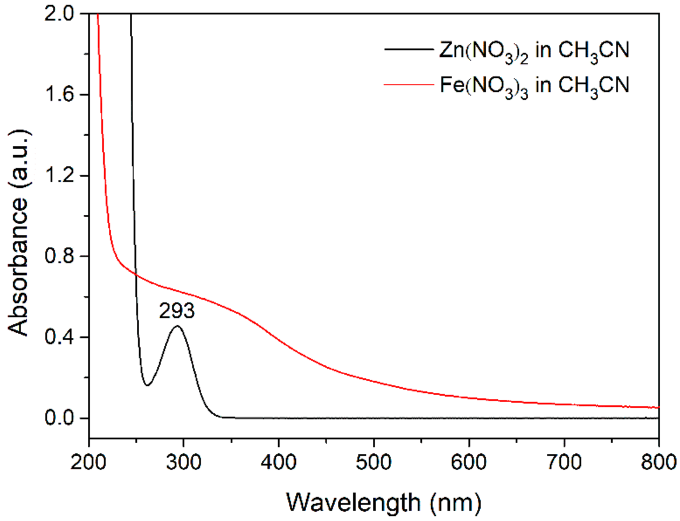

Figure S2. UV–vis spectra of $Zn(NO_3)_2$ or $Fe(NO_3)_3$ in CH_3CN solvent.

4. ATR-IR spectra in the C-N region of $Zn(NO_3)_2$/$Fe(NO_3)_3$ in CH_3CN solvent

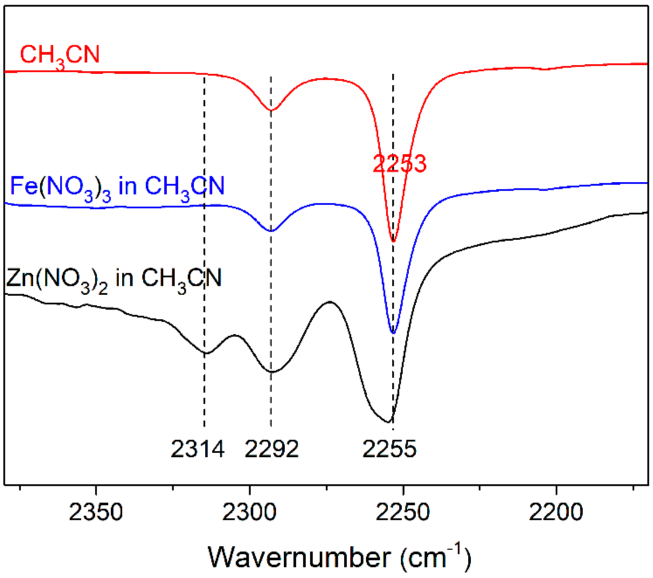

Figure S3. ATR-IR spectra in the C-N region of $Zn(NO_3)_2$/$Fe(NO_3)_3$ in CH_3CN solvent

5. The effect of catalyst loading and $Zn(NO_3)_2$ loading for catalytic oxidation of methyl DL-mandelate

The results for catalytic oxidation of methyl DL-mandelate under different catalyst loading and $Zn(NO_3)_2$ loading are shown in Table S1. Firstly, catalytic oxidation of methyl DL-mandelate with different catalyst loading was performed under the same reaction conditions (80 °C, 0.2 MPa O_2, 1.5 h). It was found that the conversion of methyl DL-mandelate increased with an increase of the catalyst loading (Table S1, Entries 1-5). The reaction in the presence of 1 mol% catalyst afforded 6% conversion of methyl DL-mandelate (Table S1, Entry 1), and the conversion of methyl DL-mandelate increased slightly even by prolonging the reaction time from 1.5 h to 7.5 h (Table S1, Entry 2). In contrast, up to 99% methyl DL-mandelate conversion with above 99% methyl phenylglyoxylate selectivity was obtained using 5 mol% catalyst (Table S1, Entry 4), and high conversion and selectivity could maintain in the presence of 10 mol% catalyst under the same reaction conditions (Table S1, Entry 5). Therefore, the optimization of catalyst loading is 5 mol%. Subsequently, the effect of $Zn(NO_3)_2$ loading for catalytic oxidation of methyl DL-mandelate was investigated under the same reaction conditions. It was found that the conversion of methyl DL-mandelate firstly increased from 6% to 99% and then decreased to 89% with an increase of the molar ratio of $Zn(NO_3)_2$/VOC_2O_4 from 0 to 4, and the selectivity toward for methyl phenylglyoxylate remained basically unchanged (Table S1, Entries 6-11). Hence, $Zn(NO_3)_2$ loading has an important effect for catalytic oxidation of methyl DL-mandelate, the optimization of molar

ratio of $Zn(NO_3)_2/VOC_2O_4$ is 1, and at this point the conversion of methyl DL-mandelate and selectivity for methyl phenylglyoxylate were more than 99% (Table S1, Entry 9).

Table S1. Oxidation of methyl DL-mandelate under different catalyst loading and $Zn(NO_3)_2$ loading. [a]

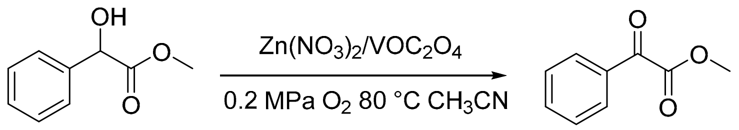

Entry	Metal nitrate (mol%)	Catalyst (mol%)	Molar ratio (nitrate/VOC_2O_4)	t (h)	Conversion (%)	Selectivity [b] (%)
1	$Zn(NO_3)_2$ (1)	VOC_2O_4 (1)	1	1.5	6	>99
2	$Zn(NO_3)_2$ (1)	VOC_2O_4 (1)	1	7.5	17	>99
3	$Zn(NO_3)_2$ (2.5)	VOC_2O_4 (2.5)	1	1.5	15	>99
4	$Zn(NO_3)_2$ (5)	VOC_2O_4 (5)	1	1.5	>99	>99
5	$Zn(NO_3)_2$ (10)	VOC_2O_4 (10)	1	1.5	>99	>99
6	-	VOC_2O_4 (5)	0	1.5	6	>99
7	$Zn(NO_3)_2$ (1.25)	VOC_2O_4 (5)	0.25	1.5	10	>99
8	$Zn(NO_3)_2$ (2.5)	VOC_2O_4 (5)	0.5	1.5	32	>99
9	$Zn(NO_3)_2$ (5)	VOC_2O_4 (5)	1	1.5	>99	>99
10	$Zn(NO_3)_2$ (10)	VOC_2O_4 (5)	2	1.5	95	>99
11	$Zn(NO_3)_2$ (20)	VOC_2O_4 (5)	4	1.5	89	>99

[a] Reaction conditions: 5 mmol methyl DL-mandelate, 5 mL CH_3CN, 80 °C, 0.2 MPa O_2, 1.5 h. [b] Selectivity toward for methyl phenylglyoxylate.

6. Catalytic oxidation of various alcohols

A wide variety of alcohol substrates such as benzylic and aliphatic alcohols were tested using $Zn(NO_3)_2/VOC_2O_4$ as catalyst under mild reaction conditions (80 °C, 0.2 MPa O_2). The summarized results for catalytic oxidation of alcohols are shown in Table S2. We are pleased to find that various benzylic alcohols such as benzyl alcohol, 4-nitrobenzyl alcohol, 2-thiophenemethanol, 2-pyridinemethanol undergo selective oxidation to afford the corresponding aldehydes with excellent yields using $Zn(NO_3)_2/VOC_2O_4$ as catalyst (Table S2, Entries 1-4). Up to 99% conversion of benzyl alcohol and 4-nitrobenzyl alcohol was transformed within 4 h, and the selectivity of benzaldehyde and 4-nitrobenzaldehyde was more than 99% (Table S2, Entries 1-2). Furthermore, nearly quantitative conversion of 2-thiophenemethanol and 2-pyridinemethanol could be facilely obtained within 1.5 h (Table S2, Entries 3-4), 84% conversion of phenylethyl alcohol with 80% selectivity of acetophenone

was obtained by prolonging the reaction time to 12 h (Table S2, Entry 5), whereas only 20% of the 3-pyridinemethanol was oxidized after 6 h (Table S2, Entry 6). Subsequently, the aliphatic alcohols are difficult to be oxidized, for the cyclohexanol and 2-amyl alcohol, 6% and 20% conversion was obtained even by prolonging the reaction time to 12 h, respectively (Table S2, Entries 7-8).

Table S2. Catalytic oxidation of various alcohols using $Zn(NO_3)_2/VOC_2O_4$. [a]

Entry	Substrate	Product	Time (h)	Conversion (%)	Selectivity (%)
1	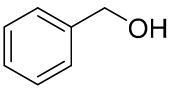	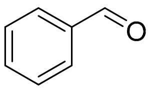	4	99	>99
2	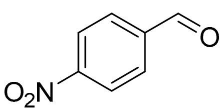	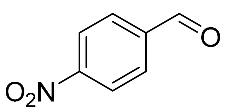	4	>99	>99
3	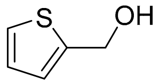	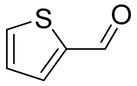	1.5	>99	>99
4[b]	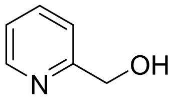	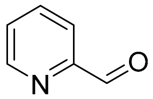	1.5	>99	90
5	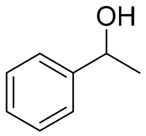	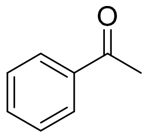	12	84	80
6	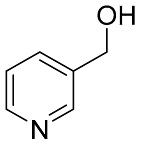	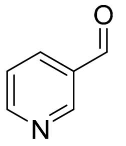	6	20	>99
7	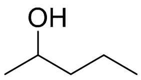	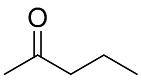	12	20	80
8	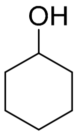	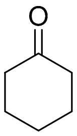	12	6	42

[a] Reaction conditions: 5 mmol substrate, 0.25 mmol $Zn(NO_3)_2$, 0.25 mmol VOC_2O_4, 5 mL CH_3CN, 80 °C, 0.2 MPa O_2. [b] 0.1 mmol $Zn(NO_3)_2$, 0.1 mmol VOC_2O_4.

7. NMR and MS spectra

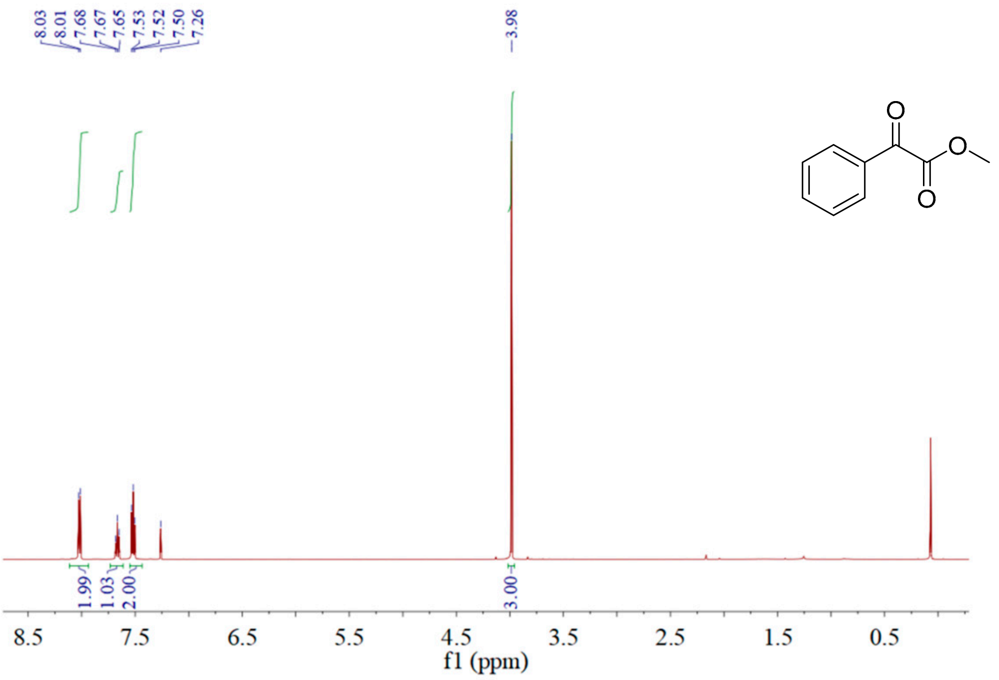

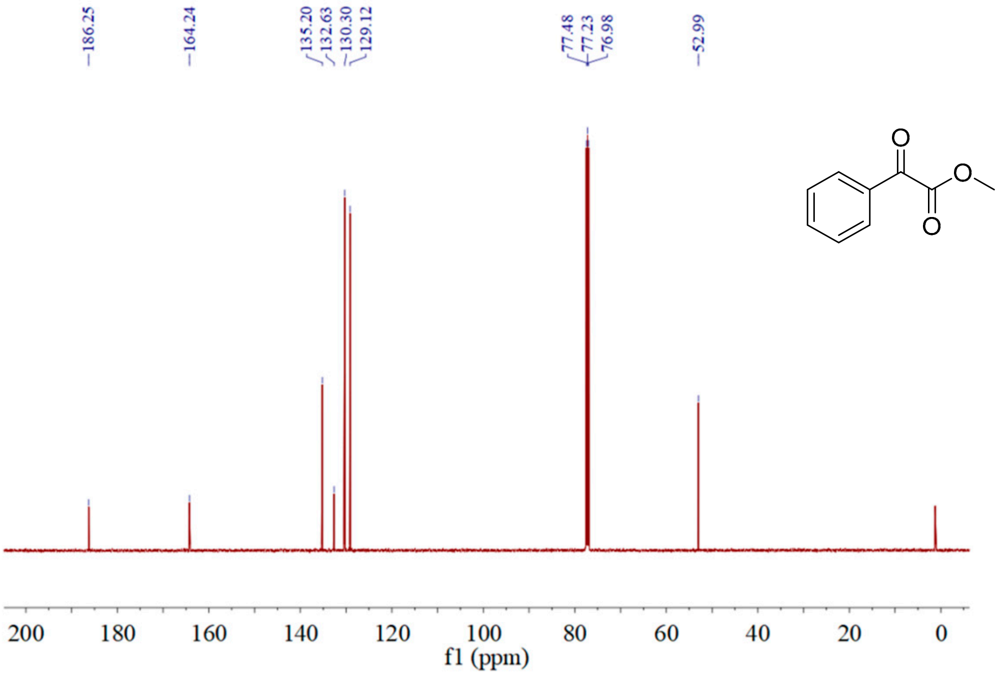

Figure S4. ^{1}H NMR and ^{13}C NMR spectra of methyl phenylglyoxylate.

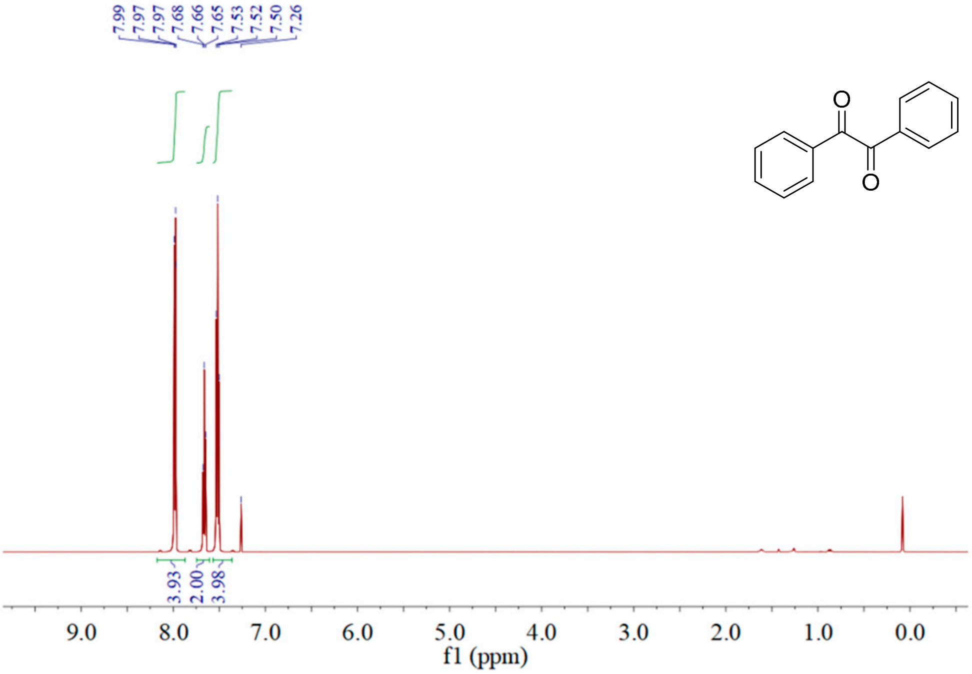

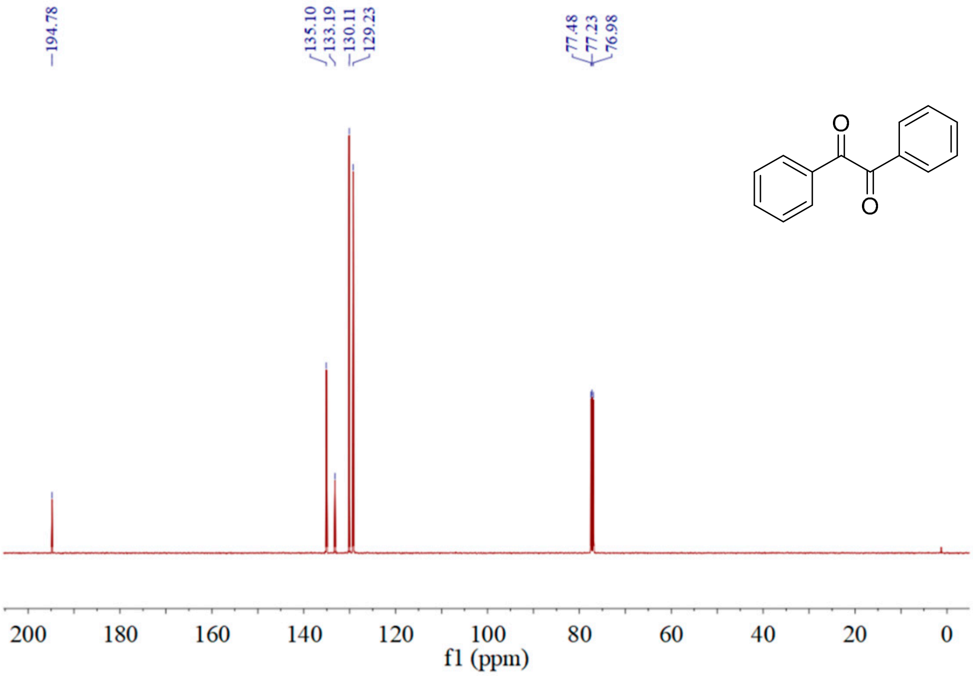

Figure S5. ^{1}H NMR and ^{13}C NMR spectra of benzil.

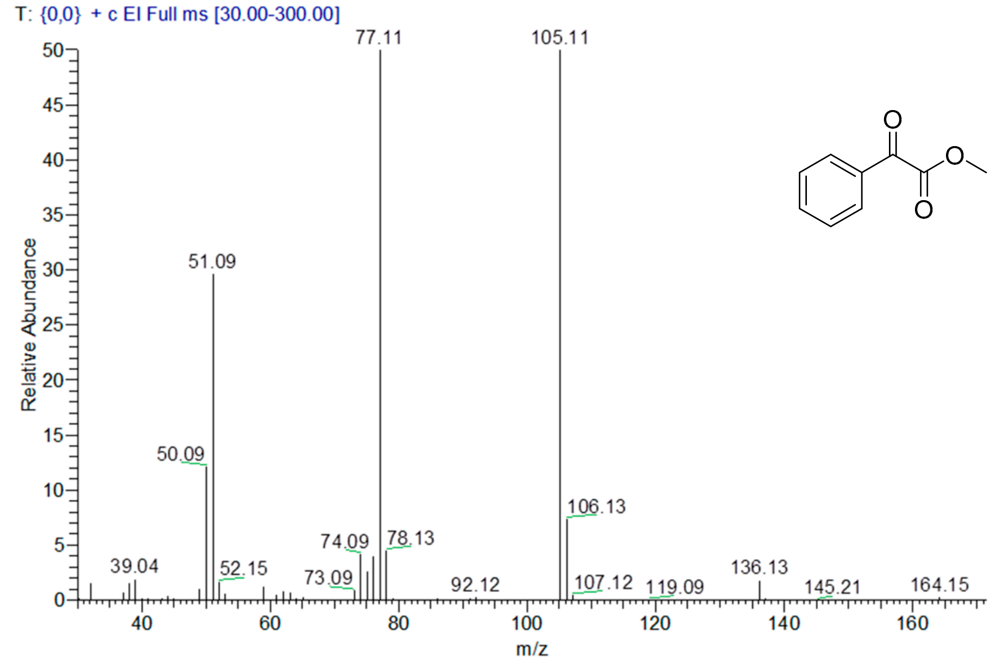

Figure S6. MS spectrum of methyl phenylglyoxylate.

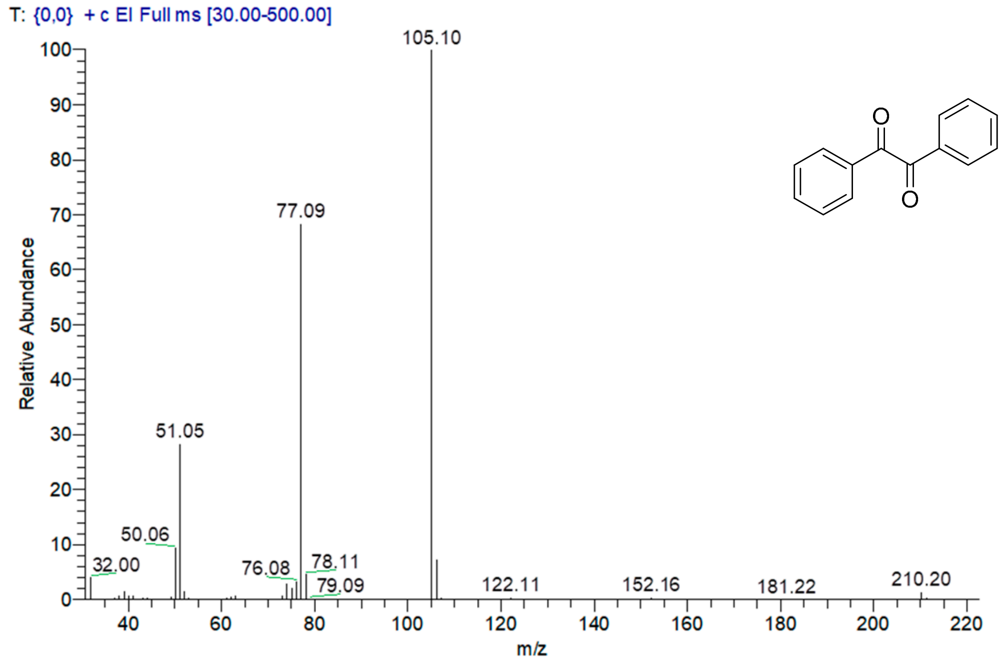

Figure S7. MS spectrum of benzil.

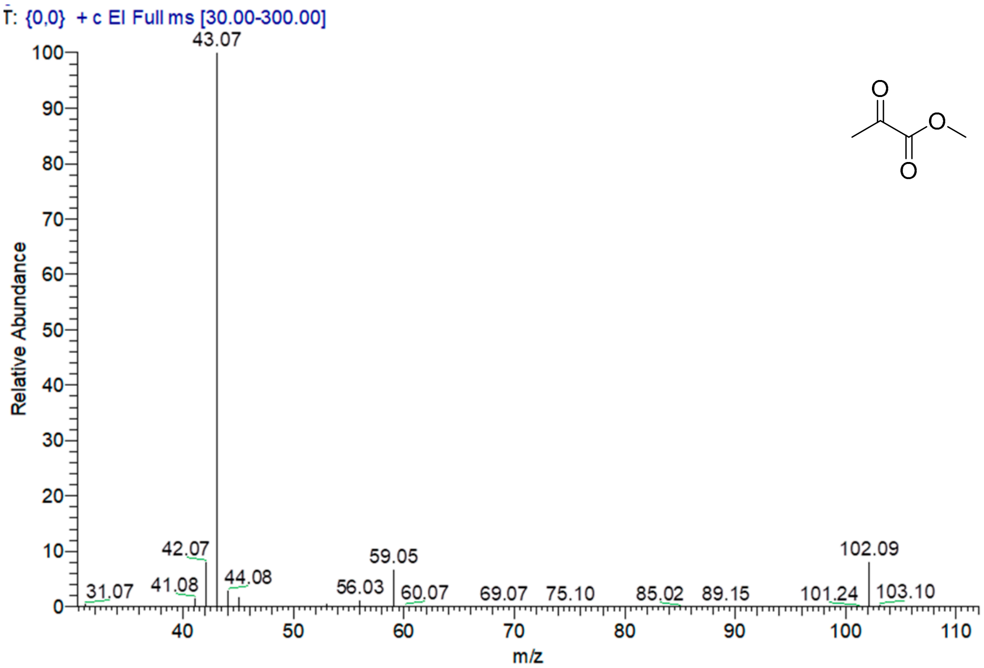

Figure S8. MS spectrum of methyl pyruvate.

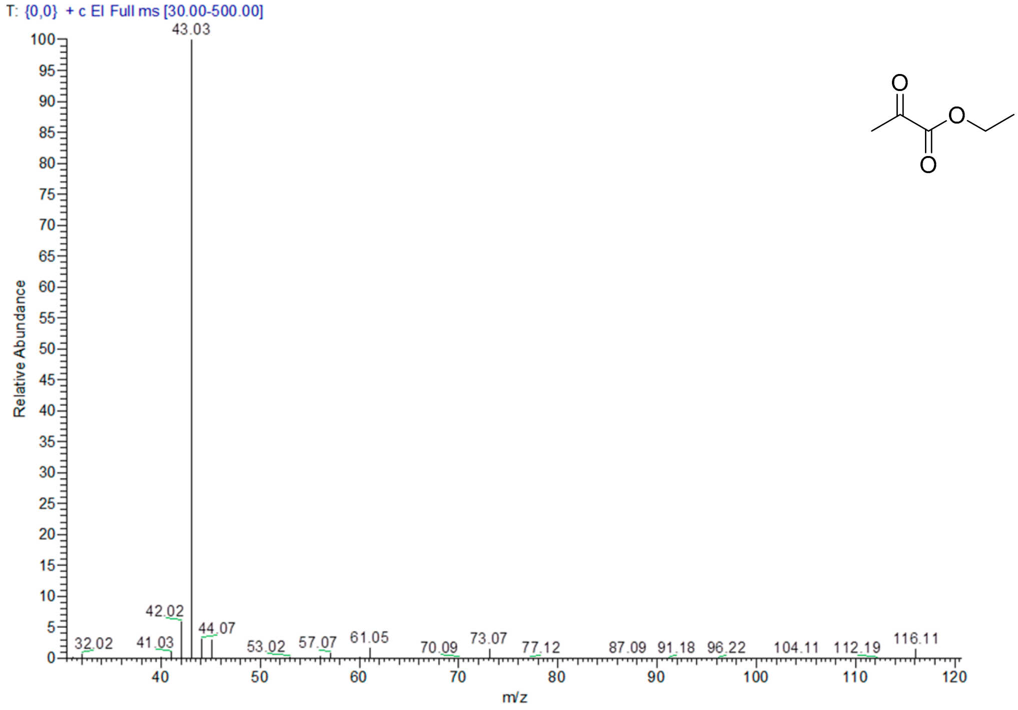

Figure S9. MS spectrum of ethyl pyruvate.

8. Original spectra of GC

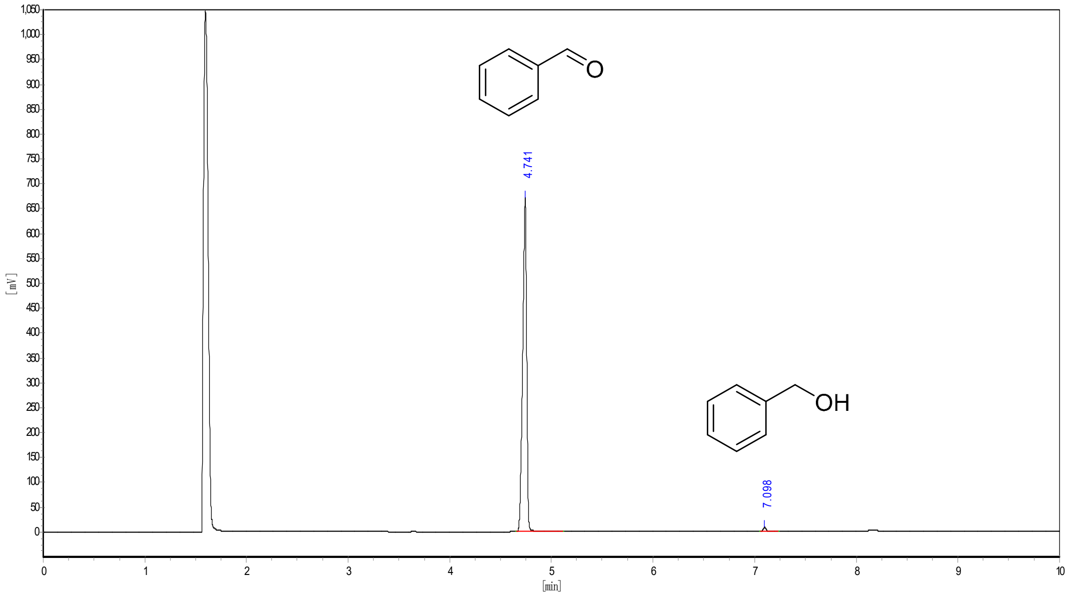

Figure S10. GC spectrum of a reaction mixture of benzyl alcohol oxidation.

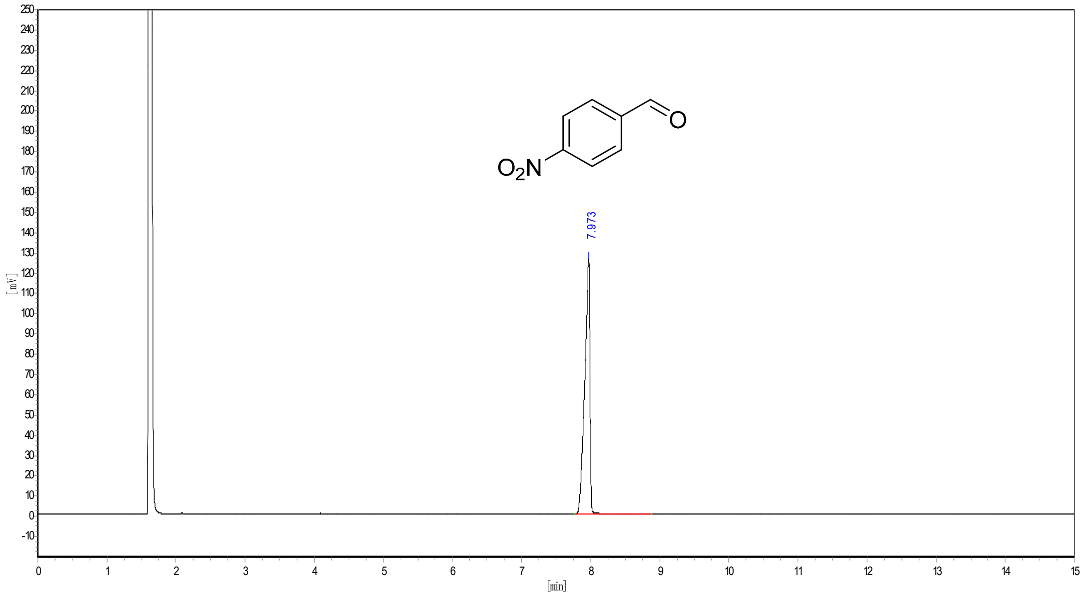

Figure S11. GC spectrum of a reaction mixture of 4-nitrobenzyl alcohol oxidation.

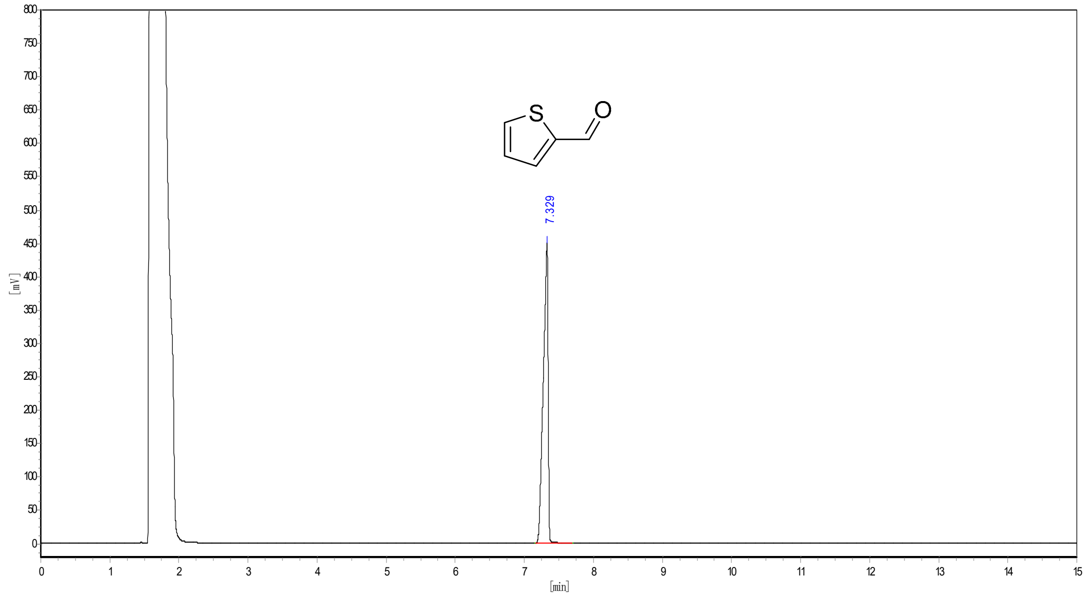

Figure S12. GC spectrum of a reaction mixture of 2-thiophenemethanol oxidation.

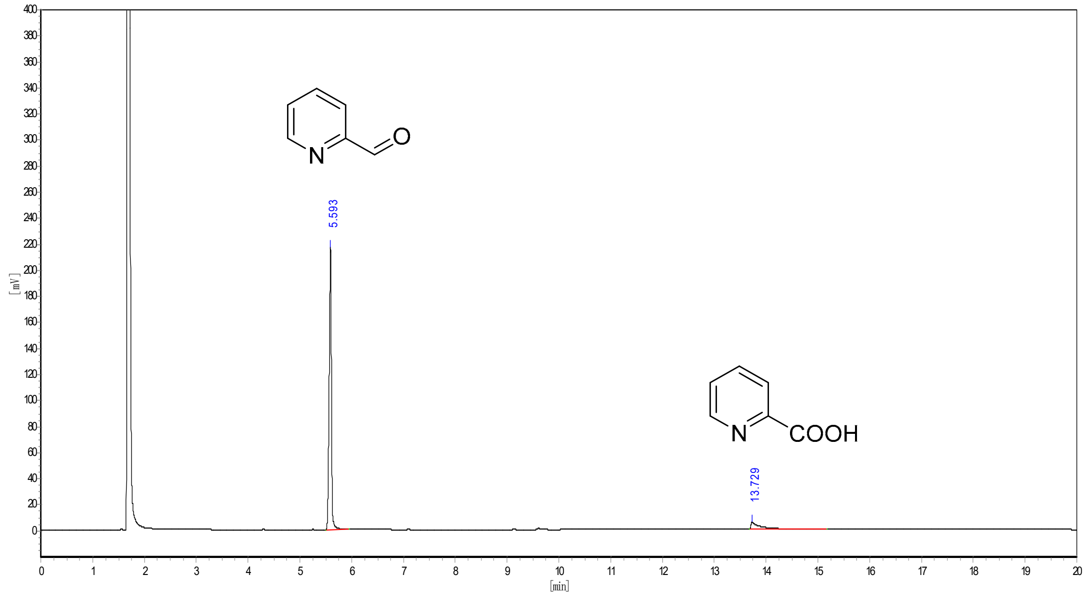

Figure S13. GC spectrum of a reaction mixture of 2-pyridinemethanol oxidation.

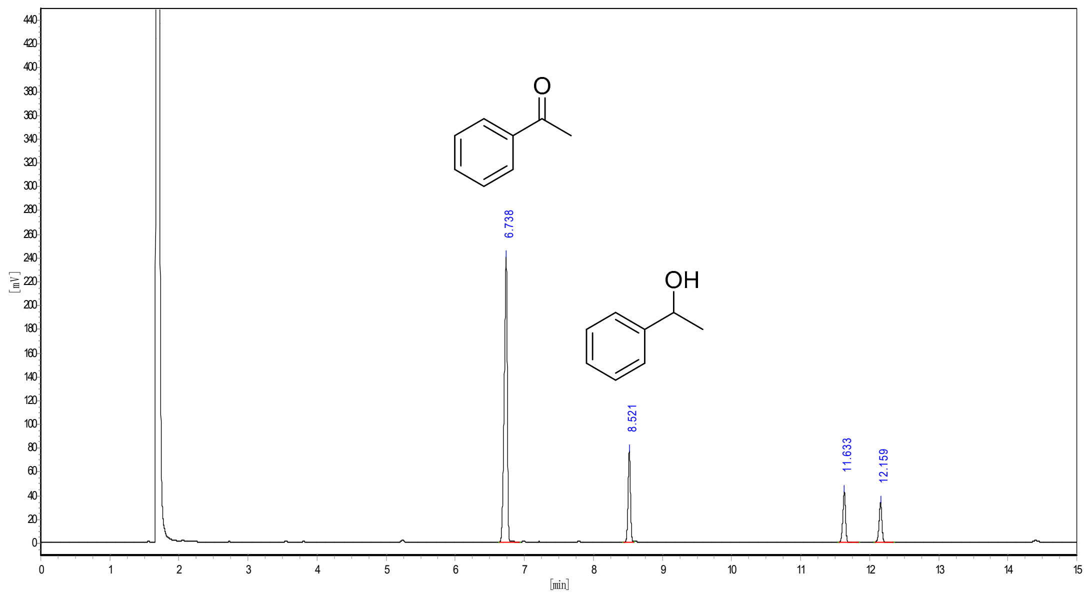

Figure S14. GC spectrum of a reaction mixture of phenylethyl alcohol oxidation.

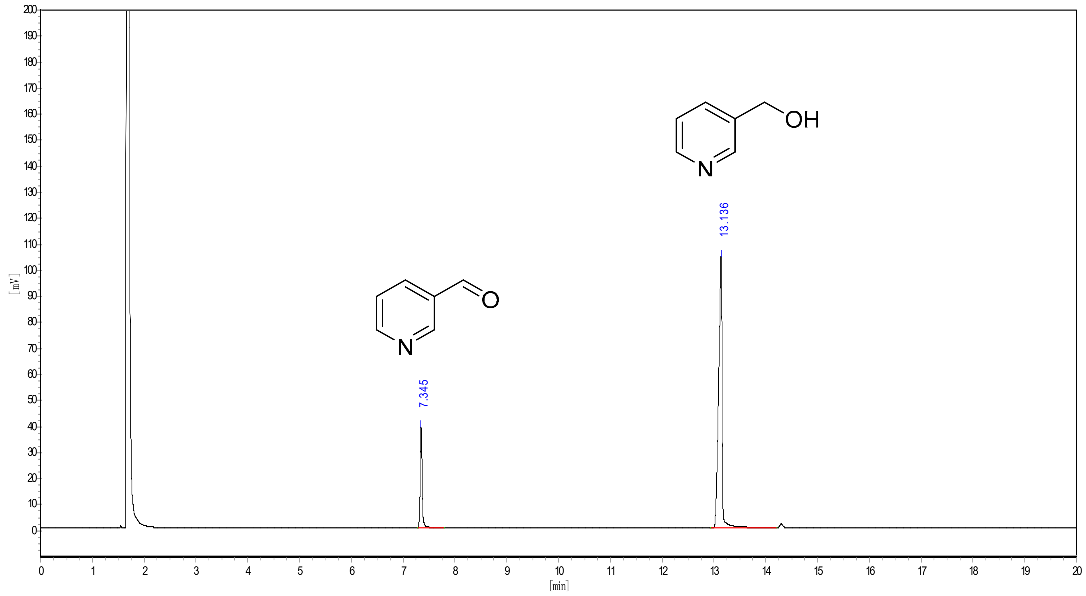

Figure S15. GC spectrum of a reaction mixture of 3-pyridinemethanol oxidation.

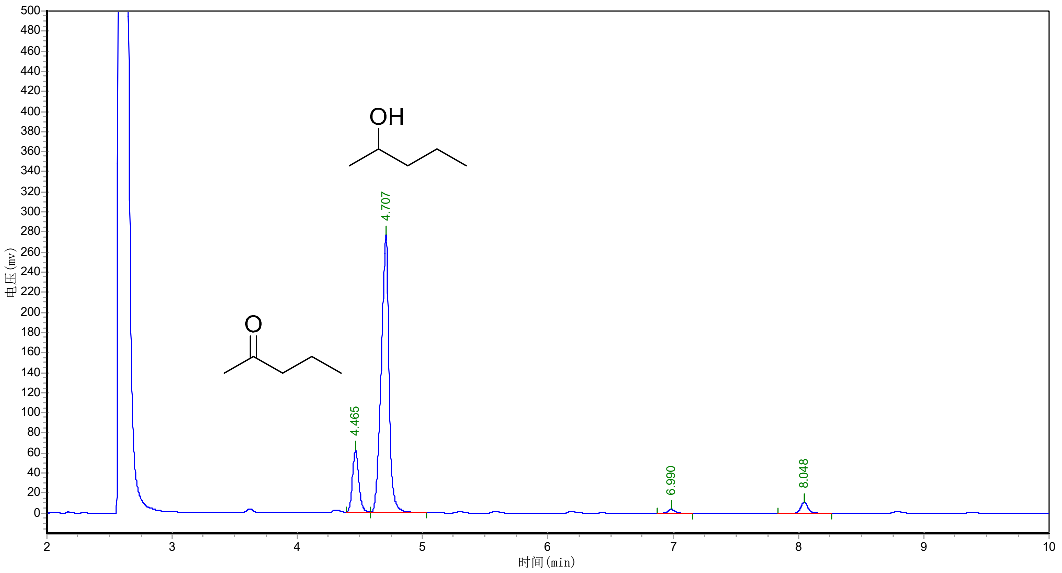

Figure S16. GC spectrum of a reaction mixture of 2-amyl alcohol oxidation.

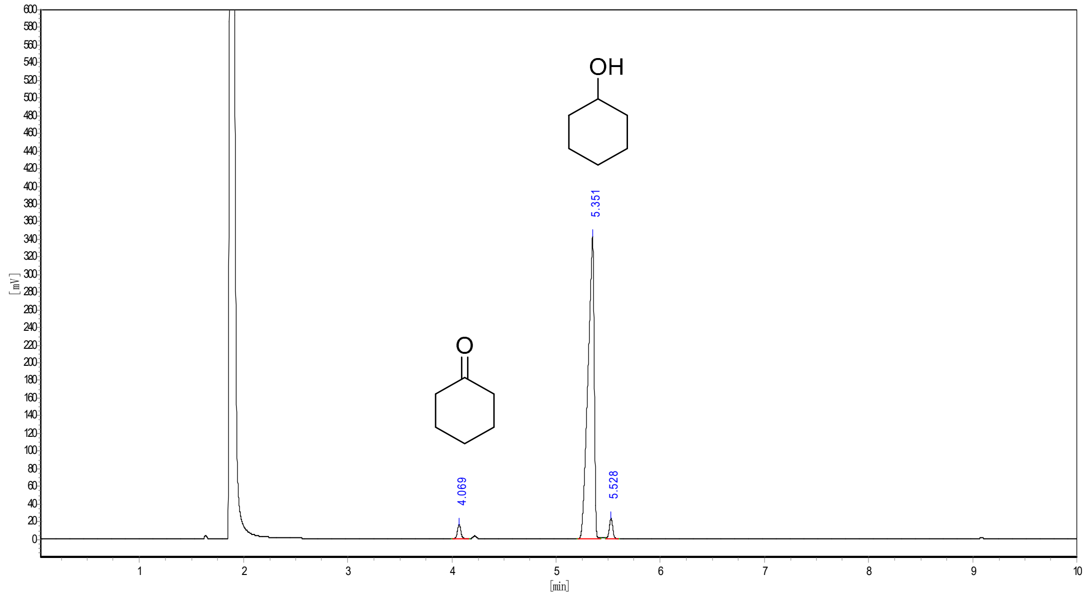

Figure S17. GC spectrum of a reaction mixture of cyclohexanol oxidation.
